# Prevalence of *Strongyloides stercoralis* and other helminths in four districts of Madagascar

**DOI:** 10.1186/s41182-024-00619-y

**Published:** 2024-07-29

**Authors:** Salvatore Scarso, Rivo Andry Rakotoarivelo, Jana Christina Hey, Tahinamandranto Rasamoelina, Anjarasoa Ravo Razafindrakoto, Zaraniaina Tahiry Rasolojaona, Nantenaina Mathieu Razafindralava, Aaron Remkes, Njary Rakotozandrindrainy, Clara Fabienne Rasoamanamihaja, Norbert Georg Schwarz, Jürgen May, Raphael Rakotozandrindrainy, Valentina Marchese, Fabio Formenti, Francesca Perandin, Francesca Tamarozzi, Cristina Mazzi, Daniela Fusco, Dora Buonfrate

**Affiliations:** 1 Department of Infectious Tropical Diseases and Microbiology, IRCCS Sacro Cuore Don Calabria Hospital, Negrar di Valpolicella, Verona, Italy; 2 University of Fianarantsoa, Fianarantsoa, Madagascar; 3 Department of Infectious Diseases Epidemiology, Bernard Nocht Institute, Hamburg, Germany; 4 German Centre for Infection Research (DZIF), Hamburg-Borstel-Lübeck-Riems, Hamburg, Germany; 5 Centre d’Infectiologie Charles Méreiux, Antananarivo, Madagascar; 6 University of Antananarivo, Antananarivo, Madagascar; 7 Ministry of Public Health, Government of Madagascar, Antananarivo, Madagascar

**Keywords:** *Strongyloides stercoralis*, Strongyloidiasis, Helminth, Prevalence study, Madagascar, *Ascaris lumbricoides*, Hookworm, *Trichuris trichiura*, *Necator americanus*, *Ancylostoma duodenale*

## Abstract

**Background:**

Estimation of prevalence of *Strongyloides stercoralis* infection is required in endemic areas, in order to identify areas in need of control programmes. Data on prevalence of strongyloidiasis in Madagascar are scant. Aim of this work was to estimate prevalence of *S. stercoralis* in four districts of Madagascar.

**Methods:**

Fecal and serum samples collected in the context of a previous study on schistosomiasis were tested with *S. stercoralis* real-time PCR and serology, respectively. A multiplex real-time PCR for *Ascaris lumbricoides*, *Ancylostoma duodenalis*, *Necator americanus*, and *Trichuris trichiura* was done on fecal samples collected in the areas demonstrating higher prevalence of strongyloidiasis. Comparisons between proportions were made using Fisher exact test, with false discovery rate correction used for post-hoc comparisons. A multivariable Firth logistic regression model was used to assess potential risk factors for *S. stercoralis* infection.

**Results:**

Overall, 1775 serum samples were tested, of which 102 of 487 (20.9%) and 104 of 296 (35.2%) were serological-positive in Marovoay and in Vatomandry districts (both coastal areas), respectively, compared to 28 of 496 (5.6%) and 30 of 496 (6.1%) in Tsiroanomandidy and in Ambositra districts (both highlands), respectively (adj. *p* < 0.001). PCR for *S. stercoralis* was positive in 15 of 210 (7.1%) and in 11 of 296 (3.7%) samples from Marovoay from Vatomandry, respectively, while was negative for all samples tested in the other two districts. High prevalence of *A. lumbricoides* (45.9%), hookworm (44.6%) and *T. trichiura* (32.1%) was found in Vatomandry. In the multivariable analysis, strongyloidiasis was associated with hookworm infection. Hookworm infection was also associated with male sex and lower education level.

**Conclusions:**

*S. stercoralis* prevalence proved higher in coastal areas compared to highlands. Different climatic conditions may explain this distribution, along with previous rounds of anthelminthics distributed in the country, which may have reduced the parasite load in the population. The high prevalence of the other soil-transmitted helminths (STH) in Vatomandry was unexpected, given the good coverage with benzimidazole in control campaigns. Further studies are needed to explore the risk factors for STH and *S. stercoralis* infections in Madagascar, in order to align with the WHO recommendations.

**Supplementary Information:**

The online version contains supplementary material available at 10.1186/s41182-024-00619-y.

## Introduction

*Strongyloides stercoralis* is a soil-transmitted helminth (STH) causing strongyloidiasis, a neglected tropical disease (NTD) widely distributed in disadvantaged areas of the world [1]. Once infected through skin penetration by infective larvae present in the soil, people develop a chronic infection that perpetuates for decades, probably lifelong, if not treated with proper anthelminthics. Strongyloidiasis often causes eosinophilia and clinical manifestations mostly involving skin, intestine and respiratory tract. Clinical manifestations can be mild and intermittent or unrelenting to severe. Notably, immunosuppression can lead to a dramatic increase in parasitic load and dissemination of the parasite all over the organism (hyperinfection/dissemination), causing a life-threatening syndrome [1].

The WHO has recommended the implementation of control activities for this infection, and integration with programmes for other NTDs is encouraged [2]. Specifically, for *S. stercoralis* a geographical overlap with the other STHs (*Ascaris lumbricoides*, hookworm, and *Trichuris trichiura*) and with intestinal schistosomiasis should be considered, due to the transmission routes entailing for all of them environmental contamination with human feces [3]. WHO guidelines for the implementation of control programmes for strongyloidiasis are being developed; in order to investigate the need for a possible intervention with ivermectin distribution, the WHO recommends to estimate the prevalence of the infection at district level.

In many countries, data on prevalence of strongyloidiasis is scarce or unavailable [4]. Kato-Katz is the recommended diagnostic test used in control programmes targeting the other STHs and schistosomiasis. Unfortunately, this technique is not suitable for diagnosis of strongyloidiasis based on an exceedingly low sensitivity for this parasite. Hence, even in areas where data on prevalence of other STHs/*Schistosoma* spp. are available, there might not be information about the geographical distribution of *S. stercoralis* [1, 4]. For the latter, the parasitological techniques with the highest sensitivity are Baermann (sensitivity usually reported in the range of 40–80%) and agar plate culture (APC, sensitivity 60–98%) [5]. These techniques are quite cheap, though require good parasitological skills to differentiate between *S. stercoralis* and other helminths’ larvae; moreover, Baermann is time-consuming and cumbersome, and for both techniques the time frame between sample collection and obtainment of the results is substantially longer (for example, at least three days for APC) than that required for Kato-Katz [5]. In addition, molecular methods (mostly, PCR) are available in referral sites, with sensitivity values similar to those reported for Baermann and APC (71.8%; 95% CI 52.2–85.5) [6]. Compared to the parasitological methods, PCR has the advantage not to need fresh, unpreserved stool: fecal samples can be preserved in ethanol or frozen for a long time before testing. However, PCR techniques lack standardization, and they are expensive compared to parasitological methods [5]. Serology has also been applied for serosurveys [7–10]. While tests based on feces are usually considered more specific than serology, in a recent study performed in an endemic area of South America, a commercial serology assay based on *S. ratti* crude antigen proved almost 100% specific in that context [11]. One of the advantages of these techniques is that the samples can be collected as dried blood spots, so that sample transportation and storage are easier. Serological assays might, however, be too expensive, and the need for cold chain and specific laboratory equipment might limit their use for control programmes. On the other hand, the combination of serology and a fecal test (parasitological or molecular) proved the diagnostic approach most performant for population screening purposes in a study conducted in Ecuador [11].

As mentioned above, ivermectin is the drug of choice for individual patient treatment as well as for public health interventions against strongyloidiasis [12]. This drug is already administered in some mass drug administration (MDA) campaigns, in combination with other anthelmintics, for the elimination of lymphatic filariasis (LF) and onchocerciasis, whereas benzimidazoles (either albendazole or mebendazole) are used in control programmes for the other STHs [12].

According to the World Bank (April 2023), in Madagascar the poverty rate is estimated to be 80.2% and approximately 77% of the population has no access to limited-standard sanitation [13]. The country is considered endemic for several NTDs including three of the five amenable to preventive chemotherapy through MDA: lymphatic filariasis (LF), schistosomiasis and STHs [14]. Although progress has been made to control LF and schistosomiasis [15, 16], prevalence of both infections within the adult population remains high, as documented in two recent studies [17, 18]. Among STHs, few surveys have assessed the impact of MDA campaigns, that were irregularly implemented and had variable coverage, and data on *S. stercoralis* in the country are particularly scant.

In this study, we took advantage of the collection of serum and fecal samples originated from the two studies on schistosomiasis mentioned above [17], to map strongyloidiasis in four districts (two coastal, two central) of the country. The aim of this study was to provide elements to support the alignment of the country with the WHO 2021–2030 NTD roadmap [2] through the identification of areas in need of interventions (i.e. MDA with ivermectin) for strongyloidiasis.

Primary objective was to estimate the prevalence of strongyloidiasis in the given districts. Secondary objective was to evaluate the association of strongyloidiasis with possible risk factors.

## Methods

Data and sample collection were described previously [17, 18]. Briefly, participants were selected from different epidemiological settings in Madagascar by sampling from primary health care centers (March 2020 to January 2021) or through a home-based survey (July to October 2022). Before the beginning of the studies, community workers were deployed to inform the communities and key community leaders about the studies. Before recruitment, all participants were informed about the study procedures through a standardized information sheet.

The study districts where samples were collected were: Marovoay (western coast), Tsiroanomandidy (highlands), Ambositra (highlands), and Vatomandry (eastern coast). From each participant, 9 ml venous blood were collected, and, if possible, a stool sample was obtained. Both serum aliquots and stool samples were stored at – 80 °C and shipped on dry ice to the Bernhard Nocht Institute for Tropical Medicine (BNITM), Hamburg, Germany. At the BNITM, available serum aliquots were selected together with all available and matching stool samples, and then shipped on dry ice to the Department of Infectious Tropical diseases and Microbiology (DITM) of IRCCS Sacro Cuore Don Calabria hospital, Negrar, Verona, Italy. There, the samples were kept at − 80 °C until analysis.

At DITM, the laboratory technicians were blinded to the areas of origin of the samples. First, the sera were tested between June and August 2023 with a commercial ELISA assay (*Strongyloides ratti* ELISA by Bordier Affinity Products, Geneve, Switzerland), following the producer indications, as described previously [11].

PCR was carried out between August and September 2023. Fecal samples were tested with singleplex in-house real-time PCR for *S. stercoralis* and, limited to the districts with high *S. stercoralis* prevalence, with a multiplex in-house real-time PCR for *Ascaris lumbricoides*, *Ancylostoma duodenalis*, *Necator americanus*, and *Trichuris trichiura*.

Genomic DNA was extracted from about 200 mg of stool, on MagEX STARlet platform (Hamilton Company, Reno, Nevada, USA) using MagMAX TM Viral/Pathogen Nucleic Acid Isolation Kit (Thermo Fisher Scientific) and the eluted DNA was used in the rt-PCR reaction. Prior to extraction, samples were spiked with a known amount of plasmid containing Phocine Herpes Virus type-1 [19], serving as an internal control for the isolation and amplification steps. The protocol for singleplex rt-PCR for *S. stercoralis* followed the one described by Verweij et al. [20]; for multiplex rt-PCR followed the one described by Llewelly et al. [21], with minor modifications. In brief, amplification reaction was performed in 25 μl volumes containing PCR buffer (SsoFast master mix, Bio-Rad Laboratories, Milan, Italy), 2.5 μg of BSA (Sigma–Aldrich), 300 nM of each of the *T. trichiura*, *A. duodenale* e *N. americanus* specific primers, 200 nM of each of di *A. lumbricoides* and 80 nM of each of the PhHV-1 specific primers, and 200 nM of *T. trichiura, A. duodenale* e *N. americanus, A. lumbricoides* labeled probe and 100 nM of PhHV-1 labeled probe.

The reactions, detection, and data analysis were performed with the CFX96 detection system (BioRad) according with the PCR protocol.

### Sample selection

Details about sample collection from the master studies have been described previously [17, 18]. A total of 500 and 1000 samples in the regions of Boeny and Atsinanana, respectively, were collected. For the present study, we tested all available sera. For areas showing high (≥ 15%) seroprevalence, all available fecal samples originating from the same individuals providing sera were tested with real-time PCR for *S. stercoralis* and for the other STHs. For those with lower seroprevalence, only fecal samples related to positive sera were tested. The threshold was chosen based on lower predictive value of serology below true prevalence 15% [22], and on the fact that for lower figures a very low number of real-time PCR would be expected to be positive.

### Data handling

Personal data originating from the previous studies were provided in a pseudo-anonymised format to the study statistician, who merged them with the diagnostic test results in an Excel file.

### Statistical analysis

For continuous variables, median and interquartile ranges (IQR) were reported. For categorical variables, frequencies and percentages were reported. Prevalence was expressed as frequency of positive tests over total number of tested samples. Prevalence based on serology and on PCR were reported separately. Comparisons between proportions were made using Fisher exact test, with false discovery rate correction used for post-hoc comparisons. A multivariable Firth logistic regression model was used to assess potential risk factors for *S. stercoralis* and other STHs positivity separately. Positive cases were defined using PCR results only. Age, sex, location, occupation, education level and other STHs infections were used as covariates. Estimates were reported as odds ratios (OR) and 95% confidence intervals (CI). *p*-values lower than 0.05 were considered significant. Analyses were performed using R software version 4.2.3.

## Results

**Fig. 1:**
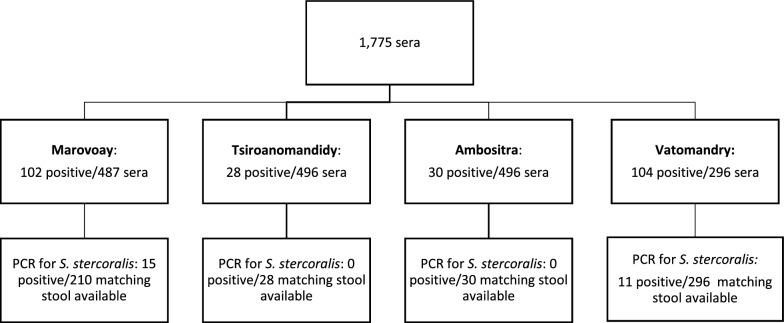
Samples analysis workflow

Figure 1 shows the workflow of the sample analyses.

A total of 1775 sera were tested, originating from a population with 54.2% female individuals, 36 years median age (min 6, max 87 years).

Coastal areas had higher prevalence of strongyloidiasis compared to the highlands. Based on serology, 20.9% and 35.2% tested samples were positive in Marovoay and in Vatomandry, respectively, compared to 5.6% and 6.1% in Tsiroanomandidy and in Ambositra, respectively (adj. *p* < 0.001).

The two coastal areas of Marovoay and Vatomandry were therefore selected for complete evaluation by PCR of all the 210 and 296 fecal samples available from each district, respectively, matching sera tested by serology, irrespective of serology results. PCR for *S. stercoralis* was positive in 7.1% samples originating from Marovoay and in 3.7% samples from Vatomandry. The results of PCR for the other STHs performed on these fecal samples are shown in Table 1.

**Table 1:** Real-time PCR results for the other STHs

Location	*N*	*A. lumbricoides* *n* (%)	*Hookworm* *n* (%)	*T. trichiura* *n* (%)
Marovoay	210	6 (2.9)	44 (21.0)	4 (1.9)
Vatomandry	296	136 (45.9)	132 (44.6)	95 (32.1)

In Tsiroanomandidy and Ambositra, fecal samples tested with PCR for *S. stercoralis*, originating only from serology-positive individuals, were all negative.

Most hookworm infections were by *N. americanus* (38/44 and 129/132 cases in Marovoay and Vatomandry, respectively).

**Fig. 2:**
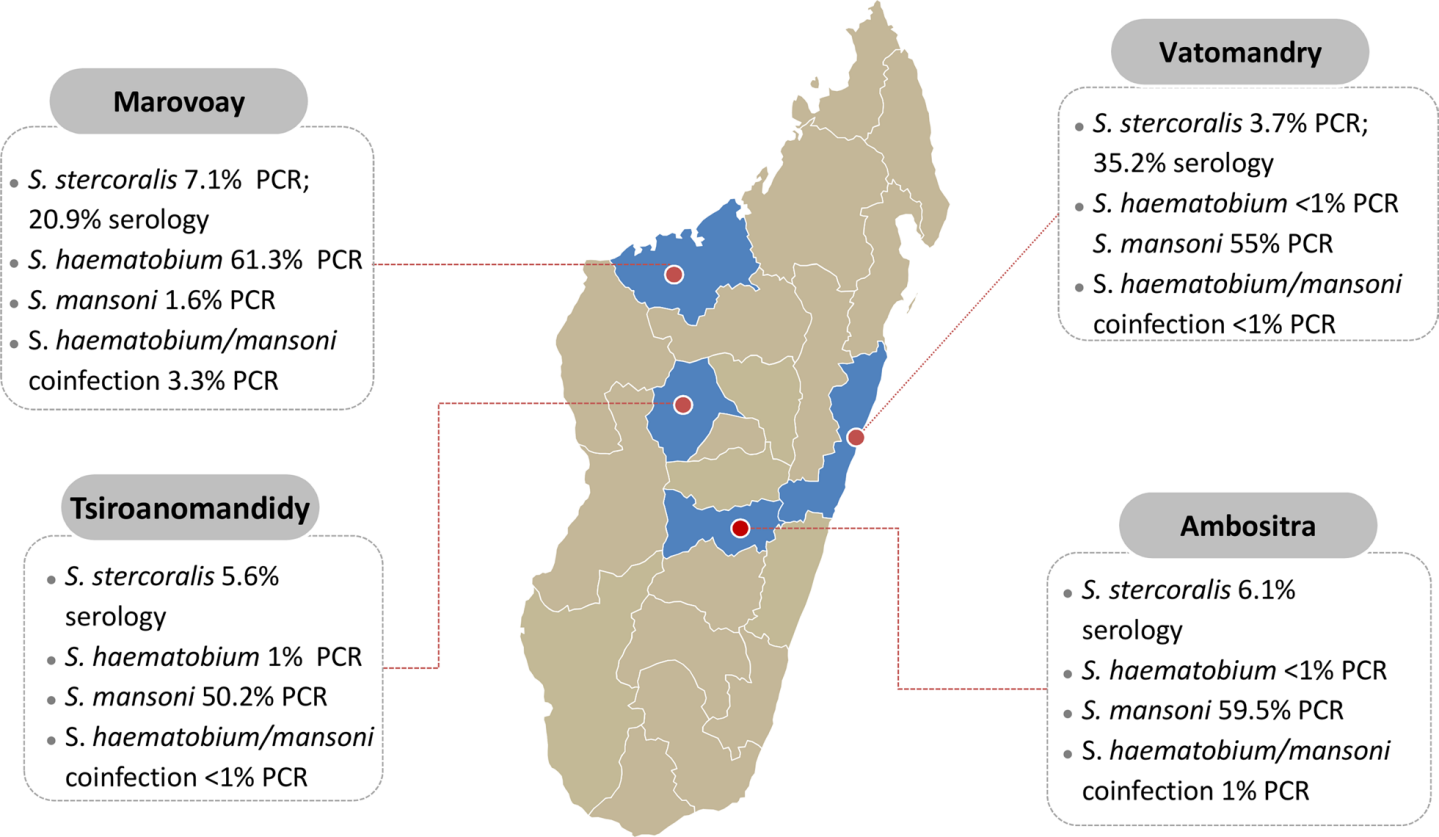
Prevalence of *S. stercoralis* in the study area by test type and overlap with schistosomiasis

Overlap of strongyloidiasis prevalence results with the distribution of schistosome infection, available from Gruninger et al. [17] is shown in Fig. 2.

In the multivariable analysis (Table 2), strongyloidiasis was associated with hookworm infection. Overall, infection with any of the other STHs was associated with infection with one or more other STHs; moreover, hookworm infection was also associated with male sex (OR: 2.59, 95% CI 1.70–4.00), and lower education level (No formal school, OR: 3.30, 95% CI 1.65–6.65) (Supplementary Table 1)*.*

**Table 2:** Results of Firth multivariable logistic regression model

Variable	OR^1^	95% CI^2^	*p*-value
Age	1.02	0.99, 1.05	0.3
Sex			
Female	–	–	
Male	1.12	0.48, 2.69	0.8
Occupation			
F armer	–	–	
Other	0.53	0.12, 1.90	0.3
Level of education			
No formal schooling	–	–	
Primary school	0.97	0.31, 3.54	> 0.9
Secondary school or higher	1.73	0.53, 6.53	0.4
Location			
Marovoay	–	–	
Vatomandry	0.11	0.02, 0.50	**0.003**
Hookworm			
Negative	–	–	
Positive	4.39	1.73, 11.8	**0.002**
*A. lumbricoides*			
Negative	–	–	
Positive	2.45	0.72, 8.75	0.2
*T. trichiura*			
Negative	–	–	
Positive	0.98	0.28, 3.11	> 0.9

Values in bold are statistically significant results

^1^OR = Odds Ratio

^2^CI = Confidence Interval

Odds ratio (OR) and 95% confidence intervals (CI) showing the association of selected factors with PCR positivity for *S. stercoralis*

## Discussion

This is the first study adopting a highly sensitive methodology to assess the prevalence of *S. stercoralis* in multiple areas of Madagascar. We found higher seroprevalence of *S. stercoralis* in the coastal areas, Marovoay and Vatomandry, compared to the highlands. In addition, we found a higher prevalence of the other STHs in Vatomandry compared to Marovoay. We did not find a clear overlap with the distribution of the different *Schistosoma* species present in the areas tested, as *S. mansoni* had a higher prevalence in the eastern coast and *S. haematobium* in the western coast.

The model confirmed that the presence of *S. stercoralis* is associated with hookworm, as it has been found previously [4]. The two parasites share a common transmission route (skin penetration by infective larvae present in contaminated soil), hence some overlap in their geographical distribution is expected [4], at least in areas where preventive chemotherapy (PC) has not changed helminth prevalence. Further studies are, however, needed for a comprehensive understanding of the mechanisms at the basis of co-infection. For the multivariate analysis, we considered *S. stercoralis* cases those with positive PCR, irrespective of the result of serology, in order to exclude possible false positive cases. Although higher prevalence was expected with serology compared to PCR, due to the higher sensitivity of the former, the discrepancy between the figures obtained with the two techniques was particularly large. Higher serology figures might be partly due to cross-reactions with other helminths. Another possible explanation is that serology can take long time to serorevert [23], so part of the positive results might mirror the distribution of previous infections.

In Madagascar, several rounds of PC for STHs were carried out with either albendazole or mebendazole since 2007 [14]. Moreover, since 2019 albendazole was distributed in association with ivermectin and diethylcarbamazine (DEC) in areas targeted for the elimination of LF [14].

It has been shown that ivermectin mass distribution, even in the context of programmes not specifically targeting *S. stercoralis*, can lead to a dramatic decrease of prevalence of strongyloidiasis [24]; in some areas, clearance from the infection or very low prevalence were maintained over several years after the end of the PC campaigns [25, 26]. In the study districts, ivermectin was not massively distributed before sample collection; however, albendazole might have reduced the parasite load in some areas (since this drug has some activity also against *S. stercoralis *[27]), affecting PCR detection capacity.

In the published literature, we found a few studies reporting prevalence of *S. stercoralis* in Madagascar. In a cross-sectional study carried out in 2016 in 12 villages of the Ifanadiana district, in central Madagascar, a prevalence of 3.3% (CI 1.84–4.77) was found with a combination of Kato-Katz and spontaneous sedimentation technique, which have low sensitivity for *S. stercoralis *[28]. Hence, we may suppose that higher prevalence could be found if a survey was carried out in the same area with more sensitive tests, such as PCR or Baermann or APC. A study carried out in 2012 in school children living in a village in the highlands of Andina township, Ambositra region, reported that one out of 410 children tested with PCR resulted positive for *S. stercoralis *[29]*.*

Overall, studies published in the literature, in addition to our data, suggest that the prevalence of strongyloidiasis is heterogenous across the country, with lower figures in central areas. This might be partly due to the presence of highlands, which might be less suitable for the external life cycle of *S. stercoralis* compared to the coastal areas, where there is milder climate. However, *S. stercoralis* transmission has been demonstrated also in highlands in other countries and in zones with colder temperatures [30].

The high prevalence of STHs in the samples from Vatomandry was unexpected, due to the repeated rounds of mebendazole with reported good coverage (at least 84%) carried out in 2018, 2020 and 2021 in the area. Though gaps in the implementation of control activities might had occurred, possible unsatisfactory drug effectiveness should also be considered. Reduced efficacy of both benzimidazole drugs has been demonstrated against *T. trichiura *[31, 32]; for hookworm, there is evidence of a reduced efficacy of mebendazole compared to albendazole [31]. For these helminths, switching from mebendazole to albendazole and introducing combination PC with ivermectin might achieve higher impact, since combination therapies have been shown to achieve better results [33, 34]. Hence, control programmes for STHs would benefit from the integration with campaigns targeting *S. stercoralis* distributing ivermectin. Different from *T. trichiura* and hookworm, however, no reduced/different efficacy of mebendazole compared to albendazole has been reported for *A. lumbricoides* [31], which was the STH with the highest prevalence in Vatomandry.

This study has some limitations. First, the number of sera tested was linked to the availability of samples retrieved from previous studies; therefore, a specific sample size calculation was not performed a-priori but post-hoc. Estimation of the sample size which could give reliable estimates of prevalence at district level leading to programme decision making, according to the accuracy of the diagnostic test (s) used is not currently available for *S. stercoralis*. If we rely on calculations made for STH [35], between 300 and 350 participants per age group would be sufficient, considering the accuracy of the serology and molecular tests used here. However, we recognize that the modeling study might not be completely applicable to strongyloidiasis, and larger surveys may be needed to confirm these data with higher precision levels. Additional districts should be included for a complete definition of *S. stercoralis* infection in Madagascar, too. However, these preliminary data can be useful to evaluate areas that need further epidemiological surveys aimed at evaluating the need for intervention. Another limitation of this study is that PCR was not carried out on all fecal samples from the highlands because the proportion of positive sera was already low, so a few samples would be expected to be positive at PCR, which is less sensitive than serology [5]. However, a low proportion of serology tests were positive among samples from the highlands, so we believe that the true prevalence should be well below the threshold eventually set by the WHO for public health intervention. Additionally, the samples were collected in the frame of studies originally designed to assess prevalence and risk associations for schistosome infections, hence the original investigation tool from which data were available fails to report critical data to assess risky behaviors typically associated to *S. stercoralis* infection (i.e. walking barefoot).

## Conclusions

In conclusion, we tested sera from four districts of Madagascar, and observed higher prevalence of *S. stercoralis* infection in the samples from the two coastal areas of Marovoay and Vatomandry. In the latter district, we also found high prevalence of the other STHs, despite the reported good PC coverage with mebendazole. Larger studies are needed to confirm these findings, and to further investigate risk factors associated with *S. stercoralis*/hookworm co-infection, the causes of high prevalence of *S. stercoralis* and other STH in specific foci, and integrated means that might improve public health control of these helminthic infections. However, our results are already suggestive for urgent the need of public health interventions in the area to align the country with the WHO NTD 2021-2030 roadmap.

### Supplementary Information

Additional file 1.

## Data Availability

The data sets analysed during the current study are available in Zenodo, at https://zenodo.org/records/12805901.

## Abbreviations

STH: Soil-transmitted helminth
NTD: Neglected tropical disease
APC: Agar plate culture
MDA: Mass drug administration
LF: Lymphatic filariasis
PC: Preventive chemotherapy
