# Supplementary material for: Prevalence of Strongyloides stercoralis and other helminths in four districts of Madagascar
Source: Trop Med Health. 2024 Jul 29;52:49. doi: 10.1186/s41182-024-00619-y (PMC11285119; doi:10.1186/s41182-024-00619-y)
Supplement: Supplementary file 1 — Additional file 1. [file 41182_2024_619_MOESM1_ESM.docx]

|  | 1. **Hookworm** | | | 1. ***Ascaris lumbricoides*** | | | 1. ***Trichuris trichiura*** | | |
| --- | --- | --- | --- | --- | --- | --- | --- | --- | --- |
|  | **OR***^1^* | **95% CI***^2^* | **p-value** | **OR***^1^* | **95% CI***^2^* | **p-value** | **OR***^1^* | **95% CI***^2^* | **p-value** |
| Age | 1 | 0.99, 1.05 | 0.3 | 1 | 0.99, 1.05 | 0.3 | 1 | 0.99, 1.05 | 0.3 |
| Sex |  |  |  |  |  |  |  |  |  |
| Female | — | — |  | — | — |  | — | — |  |
| Male | 1.1 | 0.48, 2.69 | 0.8 | 1.1 | 0.48, 2.69 | 0.8 | 1.1 | 0.48, 2.69 | 0.8 |
| Occupation |  |  |  |  |  |  |  |  |  |
| Farmer | — | — |  | — | — |  | — | — |  |
| Other | 0.5 | 0.12, 1.90 | 0.3 | 0.5 | 0.12, 1.90 | 0.3 | 0.5 | 0.12, 1.90 | 0.3 |
| Level of education |  |  |  |  |  |  |  |  |  |
| No formal schooling | — | — |  | — | — |  | — | — |  |
| Primary school | 1 | 0.31, 3.54 | >0.9 | 1 | 0.31, 3.54 | >0.9 | 1 | 0.31, 3.54 | >0.9 |
| Secondary school or higher | 1.7 | 0.53, 6.53 | 0.4 | 1.7 | 0.53, 6.53 | 0.4 | 1.7 | 0.53, 6.53 | 0.4 |
| Location |  |  |  |  |  |  |  |  |  |
| Marovoay | — | — |  | — | — |  | — | — |  |
| Vatomandry | 0.1 | 0.02, 0.50 | 0.003 | 0.1 | 0.02, 0.50 | 0.003 | 0.1 | 0.02, 0.50 | 0.003 |
| Hookworm |  |  |  |  |  |  |  |  |  |
| Negative | — | — |  | — | — |  | — | — |  |
| Positive | 4.4 | 1.73, 11.8 | 0.002 | 4.4 | 1.73, 11.8 | 0.002 | 4.4 | 1.73, 11.8 | 0.002 |
| *A. lumbricoides* |  |  |  |  |  |  |  |  |  |
| Negative | — | — |  | — | — |  | — | — |  |
| Positive | 2.5 | 0.72, 8.75 | 0.2 | 2.5 | 0.72, 8.75 | 0.2 | 2.5 | 0.72, 8.75 | 0.2 |
| *T. trichiura* |  |  |  |  |  |  |  |  |  |
| Negative | — | — |  | — | — |  | — | — |  |
| Positive | 1 | 0.28, 3.11 | >0.9 | 1 | 0.28, 3.11 | >0.9 | 1 | 0.28, 3.11 | >0.9 |
| *^1^* OR = Odds Ratio | | | | | | | | | |
| *^2^* CI = Confidence Interval | | | | | | | | | |

**Table S1.** Results of Firth multivariable logistic regression model. Odds ratio (OR) and 95% confidence intervals (CI) showing the association of selected factors with PCR positivity for (A) Hookworm**,** (B) *Ascaris lumbricoides* and (C) *Trichuris trichiura*.
